# A New Pro-Prodrug Aminoacid-Based for *Trans*-Ferulic Acid and Silybin Intestinal Release

**DOI:** 10.3390/jfb5030099

**Published:** 2014-07-24

**Authors:** Sonia Trombino, Teresa Ferrarelli, Roberta Cassano

**Affiliations:** Department of Pharmacy and Health and Nutrition Sciences, University of Calabria, 87036 Arcavacata di Rende Cosenza, Italy; E-Mails: sonia.trombino@unical.it (S.T.); teresa.ferrarelli@yahoo.it (T.F.)

**Keywords:** l-phenylalanine, silybin, *trans*-ferulic acid, lipid peroxidation, simulated gastric fluid and intestinal fluid

## Abstract

The aim of this work was the preparation and characterization of a pro-prodrug able to simultaneously transport silybin, a drug possessing various pharmacological effects, and *trans*-ferulic acid, a known antioxidant. More specifically, l-phenylalanine-*N*-(4-hydroxy-3-methoxy-phenyl) prop-2-en-*O*-(2*R*,3*R*)-3,5,7-trihydroxy-2-((2*R*,3*R*)-3-(4-hydroxy-3-methoxyphenyl)-2-(hydroxymethyl)-2,3-dihydro-benzo-(1,4)-dioxin-6-yl)croman-4-one was synthesized by using the aminoacid l-phenylalanine (l-Phe) as carrier. Indeed, l-Phe is characterized by an intrinsic chemical reactivity due to the presence of an amino group, placed on the chiral center, and of a carboxylic group. The synthesis has been characterized first by adding silybin by means of carboxylic group and then, with the aim to confer antioxidant properties to this new carrier, by linking *trans*-ferulic acid to l-Phe via amino group. The so obtained derivative was then characterized by FT-IR, and ^1^H-NMR spectroscopies. Furthermore, its ability to inhibit lipid peroxidation induced by *tert*-butyl hydroperoxide in rat liver microsomes, was evaluated. The 1,1-diphenyl-2-picrylhydrazyl radical-scavenging effect, was also assessed. The release of silybin and *trans*-ferulic acid was determined in simulated gastric and intestinal fluids over the time. The results showed that the covalent bond between both (i) silybin; or (ii) *trans*-ferulic acid and the amino acid was degraded by enzymatic reactions. In addition, the pro-prodrug, showed strong antioxidant and scavenger activities. Due to these properties, this new pro-prodrug could be applied for the treatment of intestinal pathologies and it might improve the therapeutic potential of silybin which is strongly limited by its low solubility.

## 1. Introduction

**Figure 1:**
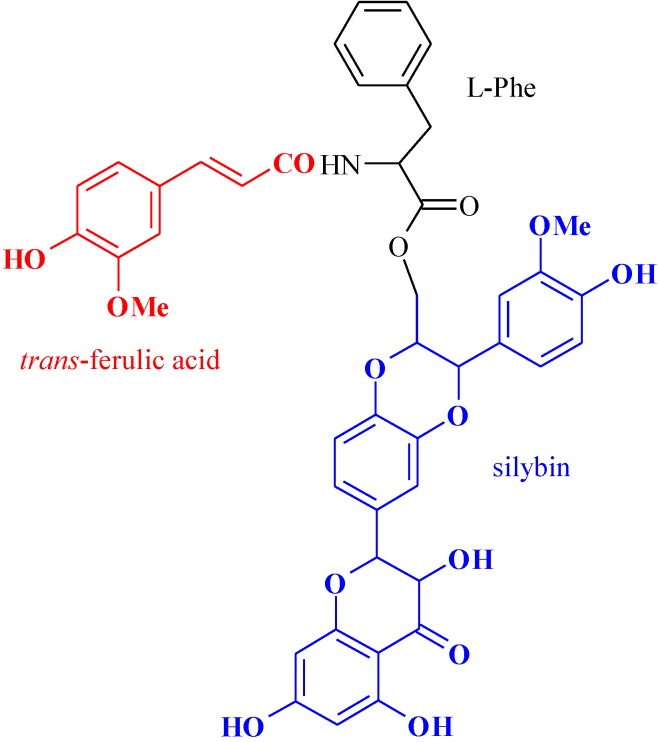
Pro-prodrug structure.

Silybin is a flavolignane isolated from the fruits of milk thistle, *Silybum marianum Gaertn*, which represents the main constituent of silymarin. Interest in this agent has grown in recent years due to its wide array of beneficial biological and pharmacological effects [1,2,3]. In particular, silybin stimulates the protein synthesis and cell regeneration [4,5] and has impressive anticancer effects against several human carcinoma cell lines [6,7]. In addition, antidiabetic activity [8], cardioprotection [9], anti-inflammatory, antifibrotic, hypolipidemic, neurotrophic, neuroprotective, and immune modulation effects have been well demonstrated [10]. Silybin possesses also antioxidant activity [11]. It was found that silybin ability to prevent free radicals-induced damage is comparable or also superior to that of vitamin E. Interestingly, it was shown that silybin containded in milk thistle stimulates superoxide dismutase (SOD) enzyme, which is a well-known free radical scavenger. Furthermore, silybin inhibits lipoxygenase, thus preventing leukotrienes synthesis and the further inflammation processes. In spite of the promising biological effects of silybin, pharmacokinetic studies performed over the past three decades and aimed to characterize silybin absorption, distribution, metabolism, and excretion have provided negative outcomes. Indeed, silybin was characterized by a (i) poor absorption; (ii) rapid metabolism; and ultimately (iii) poor oral bioavailability thus restricting its application [12,13,14]. In order to improve silybin pharmacokinetic properties and, in particular, its solubility, many efforts have been done to develop new silybin formulations, mainly based on the use of drug delivery systems, such as micro- and nano-particles and liposomes [15,16,17,18,19,20,21]. In addition, a useful approach to enhance silybin bioavailability may be its conversion into a prodrug using specific carriers [22]. Usually, amino acids are utilized since they are absolutely nontoxic. Interestingly, the simultaneous presence of at least two derivatizable and highly reactive polar groups, such as the amino (NH_2_) and carboxyl (COOH) groups, provides the possibility to produce pro-prodrugs that bind at the same time, two active substances [23,24]. In that frame, a pro-prodrug is a substrate-spacer-prodrug suited for site specific delivery of drugs. With this approach and in order to obtain a pro-prodrug with increased antioxidant properties, we also successfully bonded silybin and *trans*-ferulic acid to l-phenylalanine (Figure 1). This new pro-prodrug could be very efficient as antioxidant as well as antifibrotic, anti-inflammatory and immunomodulating agent. Finally, that formulation could be useful for liver regenerating because the release of the active substances, silybin and *trans*-ferulic acid, happen in the colon by an enzymatic cleavage [25].

## 2. Results and Discussion

In the present work, we synthesized and characterized a pro-prodrug l-phenylalanine (l-Phe) based, which simultaneously links both silybin and *trans*-ferulic acid, respectively, through an ester bond and an amido bond. Although the amides are more stable against hydrolysis, it is true that the ones derived from amino acids are much more susceptible to enzymatic hydrolysis by many amidases present in the intestine and in the colon. The covalent linking of silybin to the l-phenylalanine through an ester bond allow us to sustain the release of the active principle mediated by the esterases within the target tissue. Moreover, the pro-prodrug is sufficiently hydrophilic and hindered to minimize the absorption in the stomach and in the small intestine. 

### 2.1. Preparation and Characterization of the Pro-Prodrug

The synthesis of the pro-prodrug was carried out initially by protecting the l-Phe amino group with the purpose of reducing side reactions (Scheme I) [26,27].

The protection reaction was consequently used to obtain *N*-(*tert*-butoxycarbonyl)-l-phenylalanine (**1**) in 93% yield. Then, according to Scheme I, carboxylic function of intermediate **1** was allowed to react with the primary alcoholic function of silybin to give the ester named *N*-(*tert*-butoxycarbonyl)-l-phenylalanine-(2*R*,3*R*)-3,5,7-trihydroxy-2-((2*R*,3*R*)-3-(4-hydroxy-3-methoxyphenyl)-2-hydroxymethyl)-2,3-dihydro-benzo(1,4-dioxin-6-yl)croman-4-one (**2**) in 60% yield. Furthermore, to promote the formation of an activated ester and to support the process of esterification dicyclohexylcarbodimide (DCC) and 4-(*N*,*N*)-(dimethylamino)pyridine (DMAP) were employed. It is known that amines more easily react with DCC providing the corresponding amides. This behavior is due to higher amines nucleophilicity, compared with alcohols. Since the esterification reaction is generally slow, a side reaction that decreases the final yield and complicates the purification of the product could occur. This side reaction consists in the formation of a 1,3 *O*-acyl intermediate able to rearrange into *N*-acyl urea which is unable to further react with alcohol. In order to avoid these consequences, DMAP is added as nucleophilic catalyst.

**Scheme I:**
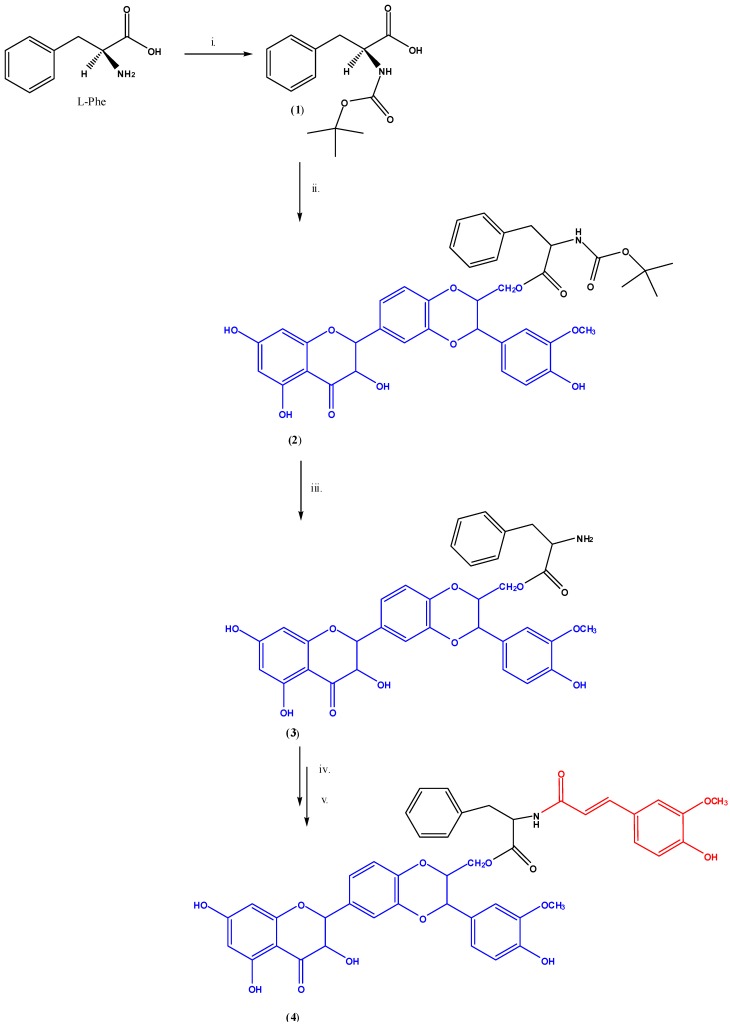
The preparation of pro-prodrug **4**.

After that, the amino group was selectively deprotected in order to synthesize the final product. The protective group –Boc_2_O, stable against most of the nucleophiles and bases, was removed in anhydrous environment and under acidic conditions, using a mixture of trifluoroacetic acid (TFA) and dichloromethane (CH_2_Cl_2_), as shown in Scheme I. As a result, the desired deprotected ester l-phenylalanine-(2*R*,3*R*)-3,5,7-trihydroxy-2-((2*R*,3*R*)-3-(4-hydroxy-3-methoxyphenyl)-2-hydroxymethyl)-2,3-dihydroxybenzo(1,4)dioxin-6-yl)croman-4-one (**3**) was obtained in 95% yield.

Finally, the amino group of compound **3** was allowed to react with the corresponding acylic chloride of *trans*-ferulic acid to give l-phenylalanine-*N*-(4-hydroxy-3-methoxy-phenyl)prop-2-en-*O*-(2*R*,3*R*)-3,5,7-trihydroxy-2-((2*R*,3*R*)-3-(4-hydroxy-3-methoxyphenyl)-2-(hydroxymethyl)-2,3-dihydro-benzo (1,4)dioxin-6-yl)croman-4-one (**4**), our pro-prodrug, in 40% yield. (Scheme I). All intermediates and the final product were characterized by FT-IR and ^1^H-NMR spectroscopies.

### 2.2. Pro-Prodrug Antioxidant Activity Evaluation

**Figure 2:**
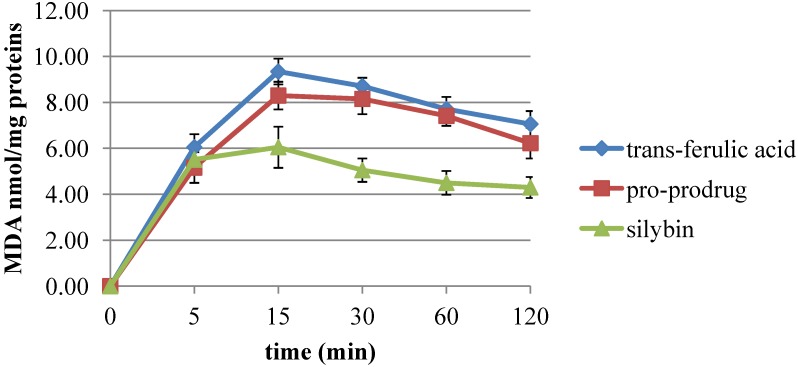
Effects of pro-prodrug on malondialdehyde (MDA) production induced by *tert*-BOOH in rat liver microsomal membranes. Microsomal membranes were incubated with 0.25 × 10^−3^ M *tert*-BOOH at 37 °C under air in the dark. The results represent means ± SEM (standard error of measurement) of six separate experiments.

The ability of pro-prodrug **4** in inhibiting *tert*-BOOH induced lipid peroxidation in rat liver microsomal membranes, during 120 minutes of incubation, was evaluated [28]. The following graph (Figure 2) shows the pro-prodrug-induced lipid peroxidation inhibition compared to both free *trans*-ferulic acid and silybin behaviors. First, these results highlight that **4** preserves the antioxidant activity of parent compounds. Then, it clearly appears that **4**-associated effect on the lipid peroxidation was time-dependent and brought as malondialdehyde (MDA) nmol/mg proteins. Furthermore, the synthesized pro-prodrug was a strong antioxidant in protecting membranes from *tert*-BOOH induced lipid peroxidation showing the preservation of activity up to 2 h.

### 2.3. Scavenger Activity of Pro-Prodrug

**Figure 3:**
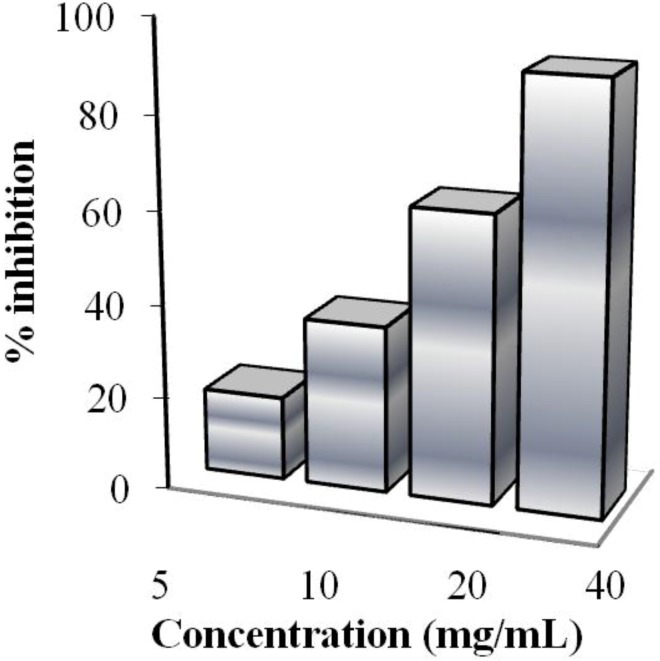
Scavenging effects of antioxidant pro-prodrug on the 2,2-diphenyl-1-picrylhydrazyl (DPPH) free radical. The results represent means ± SEM of three determinations.

The radical scavenging ability of the pro-prodrug was assessed through reaction with 2,2-diphenyl-1-picrylhydrazyl (DPPH) radicals (Figure 3). Because of its unpaired electron, the radical picrylhydrazyl shows a strong absorption band at 517 nm, assuming a purple color. When this radical is captured from an antioxidant, the absorption decreases, resulting in discoloration that is directly proportional to the number of radicals captured. For this test, a stock solution of DPPH at a known concentration is used as a control. The absorption of this solution is evaluated spectrophotometrically in the absence and presence of increasing and known amounts of the pro-prodrug. Results, reported as percentage inhibition (PI) of DPPH radicals and evaluated by the equation PI = (1-(Abs antioxidant polymers)/Abs DPPH) (Figure 3), were compared to that obtained using increasing amounts of sylibin and ferulic acid (data not shown).

### 2.4. In Vitro Release Studies of Synthesized Pro-Prodrug

The silybin and *trans*-ferulic acid release from the synthetized pro-prodrug was evaluated in SGF and SIF. The data revealed that the pro-prodrug remained intact in SGF medium. Conversely, the covalent bonds between amino acid and both silybin and *trans*-ferulic acid, were hydrolyzed in SIF medium. In particular, about 61% release of *trans*-ferulic acid, and 53% release of silybin were observed after 1 h.

*Reagents and conditions*: (i) di-tert-butyl dicarbonate (Boc_2_O), sodium bicarbonate (NaHCO_3_), tetrahydrofuran/water (THF/H_2_O), 0 °C/12 h, r. t. (25°C)/12 h; (ii) silybin, DCC, DMAP, THF, r. t., 24 h; (iii) TFA/CH_2_Cl_2_, r. t., 2h; (iv) *trans*-ferulic acid, thionyl chloride (SOCl_2_), triethylamine (Et_3_N); (vi) l-phenylalanine-(2*R*,3*R*)-3,5,7-trihydroxy-2-((2*R*,3*R*)-3-(4-hydroxy-3-methoxyphenyl)-2-hydroxymethyl)-2,3-dihydroxybenzo(1,4)dioxin-6-yl)croman-4-one (3), THF/H_2_O, r. t., 12 h.

## 3. Experimental Section

### 3.1. Materials

All solvents of analytical grade, were purchased from Carlo Erba Reagents (Milan, Italy): tetrahydrofuran (THF), *n*-hexane, chloroform (CHCl_3_), ethyl ether, dichloromethane (CH_2_Cl_2_), methanol, ethanol, triethylamine (Et_3_N). l-phenylalanine (l-Phe), di-*tert*-butyl dicarbonate (Boc_2_O), sodium bicarbonate (NaHCO_3_), dicyclohexylcarbodimide (DCC), dimethylaminopyridine (DMAP), trifluoroacetic acid (TFA), *trans*-ferulic acid, silybin, trichloroacetic acid (TCA), butylated hydroxytoluene (BHT), 2-thiobarbituric acid (TBA), *tert*-butylhydroperoxide (*tert*-BOOH), diphenyl picrilidrazile (DPPH), pancreatin, pepsin, potassium hydrogen phosphate (KH2PO4), sodium chloride (NaCl) and thionyl chloride (SOCl_2_) were purchased from Sigma-Aldrich (Sigma Chemical Co., St. Louis, MO, USA).

### 3.2. Methods

Infrared spectra were obtained from KBr pellets using a FT-IR spectrometer Perkin-Elmer 1720 (Norwalk, CT, USA), in the range 4000–400 cm^−1^ (number of scans 20). The UV-Vis spectra were realized using a UV-530 JASCO spectrophotometer. ^1^H-NMR spectra were processed using a Bruker VM300 ACP spectrometer (Billerica, MA, USA); chemical shifts are expressed in δ and referred to the solvent. Thin layer chromatography (TLC) was performed on polygram Sil G/UV254 silica gel sheets. Preparative column chromatography employed silica gel (Merck Kieselgel 60, 70–230 mesh, Merck KGaA, Darmstadt, Germany). Simulated intestinal fluid (SIF), consisting of 10 mg·mL^−1^ pancreatin (Sigma-Aldrich) in 50 mM KH_2_PO_4_ at pH 7.2, and simulated gastric fluid (SGF), consisting of 3.2 mg/mL pepsin in 0.03 M NaCl at pH 1.2, were prepared as described in the United States Pharmacopoeia (2004) [29].

### 3.3. Synthesis of N-(Tert-Butoxycarbonyl)-l-phenylalanine (1)

This reaction was conducted according to the procedure reported in the literature [26]. Briefly, 300 mg (1.82 × 10^−3^ mol) of l-Phe, 459 mg (5.46 × 10^−3^ mol) of NaHCO_3_, 0.50 mL (2.18 × 10^−3^ mol) of Boc_2_O were dissolved in a mixture of dry THF and H_2_O (1:1). The reaction mixture was left under magnetic stirring at 0 °C for 12 h and, then, at room temperature overnight. The reaction was monitored by TLC using chloroform as eluent. The mixture was dried under reduced pressure to yield an oil that, washed with THF, furnished a white solid (**1**) in 93% yield. This was analyzed by FT-IR spectroscopy and ^1^H-NMR. FT-IR (cm^−1^): 1694 (–CONH). ^1^H-NMR (D_2_O) δ (ppm): 7.0–7.15 (m, 5H), 3.35 (tb, 1H), 1.90–2.10 (m, 2H), 1.10 (s, 9H).

### 3.4. Synthesis of N-(Tert-Butoxycarbonyl)-l-phenylalanine-(2R,3R)-3,5,7-Trihydroxy-2-((2R,3R)-3-(4-Hydroxy-3-Methoxyphenyl)-2-Hydroxymethyl)-2,3-Dihydro-Benzo(1,4)Dioxin-6-yl)Croman-4-One (2) [27]

*N*-(*tert*-butoxycarbonyl)-l-phenylalanine (**1**, 100 mg, 3.77 × 10^−4^ mol) was added to a solution of silybin (186 mg, 3.77 × 10^−4^ mol) in dry THF (50 mL). The solution was stirred for 10 minutes and subsequently added of DMAP (23 mg, 1.88 × 10^−4^ mol) and DCC (106 mg, 4.90 × 10^−4^ mol). The mixture was stirred at 50 °C to enhance DCC dissolution (1 h) then left under stirring at room temperature for 24 h. The reaction was monitored by TLC using a mixture of methanol/triethylamine (9.5/0.5) as eluent. The raw product, because of its intrinsic instability, was purified only by washing with *n*-hexane and diethyl ether furnishing **2** in 60% yield that was analyzed by FT-IR and ^1^H-NMR spectroscopies. FT-IR (cm^−1^): 3013 (aromatic CH), 1733 (C=O ester), 1717 (C=O ketone), 1684 (–CONH). ^1^H-NMR (C_2_D_6_SO) δ (ppm): 6.50–7.0 (m, 13H), 4.85 (d, 1H), 4.63 (d, 1H), 4.20 (d, 1H), 3.80 (s, 3H), 3.55 ( m, 1H), 3.30 (m, 2H), 3.20 (tb, 1H), 2:25 to 2:40 (m, 2H), 1.15 (s, 9H).

### 3.5. Synthesis of l-phenylalanine-(2R,3R)-3,5,7-Trihydroxy-2-((2R,3R)-3-(4-Hydroxy-3-Methoxyphenyl)-2-Hydroxymethyl)-2,3-Dihydroxybenzo(1,4)Dioxin-6-yl)Croman-4-One (3)

*N*-(*tert*-butoxycarbonyl)-l-phenylalanine-(2*R*,3*R*)-3,5,7-trihydroxy-2-((2*R*,3*R*)-3-(4-hydroxy-3-methoxyphenyl)-2-hydroxymethyl)-2,3-dihydro-benzo(1,4)dioxin-6-yl)croman-4-one (**2**, 235 mg, 4.22 × 10^−4^ mol) was added to TFA/CH_2_Cl_2_ in a molar feed ratio of 1:1. The reaction mixture was left stirring at room temperature for 2 h. After that, the solvent was removed under reduced pressure to yield a crude product that was purified by washing with *n*-hexane and, then, with diethyl ether and THF. The solid obtained (**3**) was filtered, dried under reduced pressure and characterized by FT-IR ^1^H-NMR. FT-IR (cm^−1^): 3013 (aromatic CH), 1736 (C=O ester), 1728 (C=O ketone). ^1^H-NMR (C_2_D_6_CO) δ (ppm): 6.65–7.60 (m, 13H), 4.92 (d, 1H), 4.55 (d, 1H), 4.0 (d, 1H), 3.83 (s, 3H), 3.50 (m, 1H), 3.40 (m, 2H), 3.20 (m, 1H), 2:35 to 2:50 (m, 2H).

### 3.6. Synthesis of l-phenylalanine-N-(4-hydroxy-3-methoxy-phenyl)prop-2-en-O-(2R,3R)-3,5,7-trihydroxy-2-((2R,3R)-3-(4-hydroxy-3-methoxyphenyl)-2-(hydroxymethyl)-2,3-dihydro-benzo (1,4)dioxin-6-yl)croman-4-one (4)

*Trans*-ferulic acid (37.2 mg, 1.91 × 10^−4^ mol), SOCl_2_ (0,021 mL, 2.89 × 10^−4^ mol) and Et_3_N (0.040 mL, 2.86 × 10^−4^ mol) were dissolved in 20 mL of dry THF. The reaction mixture was left at room temperature for 6 h and then added of deprotected ester (**3**, 120 mg, 1.91 × 10^−4^ mol) in order to obtain the corresponding amide (**4**). The reaction mixture was monitored for 12 h by TLC using as eluent mixture CHCl_3_/methanol (1:1). After this time, the solvent was evaporated under reduced pressure to give the product which was loaded on a silica gel column chromatography and eluted with CHCl_3_/methanol (1:1) to yield **4** (40%) after solvent evaporation . FT-IR (cm^−1^): 3013 (aromatic CH), 1759 (C=O ester), 1736 (C=O ketone), 1681 (–CONH). ^1^H-NMR (C_2_D_6_CO) δ (ppm): 6:37 to 7:28 (m, 16H), 6.95 (d, 1H), 6.40 (d, 1H), 4.85 (d, 1H), 4.55 (m, 1H), 4.10 (m, 1H), 3.80 (s, 3H), 3.75 (s, 3H), 3.62 (m, 1H), 3.40 (m, 2H), 3.25 (m, 1H), 2.55–2.62 (m, 2H).

### 3.7. Inhibition of Lipid Peroxidation in Rat Microsomal Membranes

In order to evaluate the antioxidant activity, pro-prodrug **4** was submitted to studies, aimed at the evaluation of malondialdehyde (MDA) formation, using rat liver microsomal membranes [30]. These, being make up of phospholipids, with a high content of polyunsaturated fatty acids are an ideal substrate for the lipid peroxidation process. During this reaction, in fact, fatty acids are converted into toxic metabolites such as aldehydes. In particular, malondialdehyde is generated in a costant manner and acts as a good indicator of peroxidation rat [31]. In order to simulate the process of peroxidation, a pro-oxidant agent, such as *tert*-butylhydroperoxide (*tert*-BOOH), was used to catalyze the formation of hydroxyl radicals (•OH) responsible of this process. Microsomal membranes were incubated in the presence of *tert*-BOOH (0.25 mM) in air and in the dark. Low concentrations of MDA in the presence of the final product (4) is an index of its protective effect. Liver microsomes were prepared from Wistar rats by tissue homogenization with 5 volumes of a fresh solution containing sucrose 0.25 M, Hepes 5 mM, EDTA 0.5 mM, at pH 7.5 and in a Potter-Elvehjem homogenizer. Microsomal membranes were isolated by removal of the nuclear fraction at 3000 rpm for 10 minutes and the mitochondrial fraction at 7000 rpm for 10 min. The microsomal fraction was sedimented at 40,000 rpm for 60 min, washed once with 0.15 M HCl, collected and centrifuged again at 40,000 rpm for 30 min [32]. The membranes were suspended in 0.1 M phosphate buffer at pH 7.5 and stored at −80 °C. The protein concentration was determined using the Bio-Rad method [33]. Microsomes were suspended in 0.1 M phosphate buffer at pH 7.5. Product **4**, silybin, and *trans*-ferulic acid were added rapidly and separately to microsomes. The microsomes were added to the amount of *tert*-BOOH to reach a final concentration of 0.25 mM. Microsomal suspension, gently suspended using a homogenizer, were incubated at 37 °C under agitation under air in the dark for 1 h. Subsequently, aliquots of 1 mL of microsomal suspension (0.5 mg of proteins) were added to a solution consisting of 3 mL of 0.5% trichloroacetic acid (TCA), 0.5 mL thiobarbituric acid (TBA) (two parts 0.4% TBA in 0.2 M HCl and one part distilled water) and 0.07 mL of 0.2% butylated hydroxytoluene (BHT) in 95% ethanol. Samples were then incubated in a bath at 90 °C for 45 min and then centrifuged. After incubation, the TBA-MDA complex was extracted with 3 mL of isobutyl alcohol and detected by UV-Vis spectrophotometry at 535 nm.

### 3.8. Evaluation of the Antiradical Activity Using DPPH Test

The ability of the prodrug to act as a radical scavenger was considered. Its radical scavenging ability was assessed through the reaction with stable DPPH radicals, using the methodology of Wang *et al.* (1998) [34]. DPPH typically extracts a proton to form DPPH during the reaction [35]. Briefly, in an ethanol solution of DPPH radical (final concentration 8 × 10^−5^ M), pro-prodrug was added, and the solution concentrations were 5, 10, 20, and 40 mg/mL. The reaction mixtures were soaked vigorously and then kept in the dark for 30 min. Their absorbances were measured in 1 cm cuvettes using a UV-Vis spectrophotometer (V-530 JASCO, Halifax, Nova Scotia, Canada) at 517 nm against a blank, in which DPPH was absent. All tests were run in triplicate and averaged.

### 3.9. In Vitro Hydrolysis Studies of Synthesized Pro-Prodrug

*In vitro* hydrolysis studies of synthesized pro-prodrug were carried out in simulated gastric fluid (SGF) and in simulated intestinal fluid (SIF) [36]. In particular, a solution of 10 mg of pro-prodrug was prepared in 90 mL of SIF (pH 7.2) or SGF (pH 1.2). An aliquot of 15 mL of this solution was withdrawn repeatedly and kept in test tubes maintained at 37 ± 0.5 °C. At 1 h an aliquot was withdrawn from test tubes and was transferred to microcentrifuge tubes, followed by the addition of THF to make up the volume. The tubes were placed in freezing mixture, in order to arrest further hydrolysis, followed by vortexing at high speed for 5 min. After vortexing, the tubes were centrifuged at high speed (3000 rpm) for 5 min. 5 mL of clear supernatant, obtained from each tube, was measured by a UV-530 JASCO spectrophotometer to evaluate the amount of free silybin and *trans*-ferulic acid, released after the hydrolysis of pro-prodrug in SGF or SIF and at 288 nm and 320 nm, respectively.

## 4. Conclusions

The aim of this work was to synthesize and characterize a pro-prodrug, l-Phe based, which simultaneously carry two active substances, *i.e.*, silybin and *trans*-ferulic acid. l-Phe is the essential precursor of levodopa and catecholamines, adrenaline and noradrenaline; its use as carrier is due to the total biocompatibility and non-toxicity. Additionally, the presence on its structure of multiple functionalizable groups, such as carboxylic and aminic, allowed to design a pro-prodrug for the intestinal-specific release of two active molecules. By using this approach, silybin, also known for its anticancer action, and trans-ferulic acid, possessing remarkable antioxidant properties, were linked to l-Phe. Indeed, the pro-prodrug showed strong antioxidant and scavenger activities. In addition, it was hydrolized in a simulated intestinal environment, thus reinforcing the concept that this carrier is able to release either silybin or *trans*-ferulic acid. For these reasons the pro-prodrug could be used in the treatment of various diseases especially regarding the intestinal pathologies.
